# Prenatal evaluation in primary care in Northeast Brazil: factors associated with its adequacy

**DOI:** 10.11606/S1518-8787.2019053001024

**Published:** 2019-04-26

**Authors:** Esther Pereira da Silva, Antônio Flaudiano Bem Leite, Roberto Teixeira Lima, Mônica Maria Osório

**Affiliations:** I Universidade Federal de Pernambuco. Programa de Pós-Graduação em Nutrição. Recife, PE, Brasil; II Universidade Federal de Pernambuco. Centro acadêmico de Vitória de Santo Antão. Faculdade de Nutrição. Departamento de Nutrição. Vitória de Santo Antão, PE, Brasil; III Universidade Federal da Paraíba. Faculdade de Nutrição. Departamento de Nutrição. João Pessoa, PB, Brasil; IV Universidade Federal de Pernambuco. Faculdade de Nutrição. Departamento de Nutrição. Recife, PE, Brasil

**Keywords:** Prenatal Care, organization & administration, Health Services Coverage, Outcome and Process Assessment (Health Care), Health Status Disparities, Cuidado Pré-Natal, organização & administração, Cobertura de Serviços de Saúde, Avaliação de Processos e Resultados (Cuidados de Saúde), Disparidades nos Níveis de Saúde

## Abstract

**OBJECTIVE:**

To characterize prenatal care and verify possible factors associated with its adequacy.

**METHODS:**

This is a cross-sectional study based on interviews with health care professionals and consultations on official documents of women attending prenatal of the primary health care in the city of João Pessoa, capital of Paraíba, in the Northeast region of Brazil. Prenatal care was evaluated by an index with criteria referring to aspects of structure, process and outcome, denominated IPR/Prenatal. The multivariate logistic regression method revealed that demographic, socioeconomic, reproductive and maternal morbidity variables were possible determinants for prenatal adequacy.

**RESULTS:**

The survey involved 130 services and 1,625 primary health care patients. Prenatal care was adequate in approximately 23% of the cases. Low prevalence of referral to maternity, educational strategies and examinations were observed. The analysis showed that non-adolescent women (OR = 1,390), with a longer period of schooling (OR = 1.750), higher *per capita* income (OR = 1,870) and primiparous women (OR = 1,230) were more likely to have an adequate prenatal.

**CONCLUSIONS:**

Prenatal care, when evaluated by broader criteria, showed a low percentage of adequacy. Strategies should be developed to ensure the referral to the maternity where the birth will take place and health education activities and examinations to provide adequate prenatal care in the municipality under study. In addition, factors associated with adequacy must be considered by managers and health professionals.

## RESUMO

**OBJETIVO:**

Caracterizar a assistência pré-natal e verificar possíveis fatores associados à sua adequação.

**MÉTODOS:**

Este é um estudo transversal realizado a partir de entrevistas com os profissionais de serviços de saúde e de consultas em documentos oficiais de mulheres assistidas no pré-natal da atenção primária à saúde na cidade de João Pessoa, capital da Paraíba, na região Nordeste brasileira. O pré-natal foi avaliado por meio de um índice com critérios referentes aos aspectos de estrutura, processo e resultado, denominado IPR/Pré-Natal. Verificou-se pelo método de regressão logística multivariada se variáveis demográficas, socioeconômicas, reprodutivas e de morbidades maternas foram possíveis fatores determinantes para a adequação do pré-natal.

**RESULTADOS:**

A pesquisa envolveu 130 serviços e 1.625 pacientes da atenção primária de saúde. O pré-natal se apresentou adequado em aproximadamente 23% dos casos. Foram observadas baixas prevalências de encaminhamento à maternidade, estratégias educativas e realização de exames. As análises mostraram que mulheres não adolescentes (OR = 1,390), com maior tempo de escolaridade (OR = 1,750), maior renda *per capita* (OR = 1,870) e primíparas (OR = 1,230) apresentaram maiores chances de ter um pré-natal adequado.

**CONCLUSÕES:**

O pré-natal, ao ser avaliado por critérios mais amplos, mostrou baixo percentual de adequação. Devem ser elaboradas estratégias que garantam o encaminhamento para a maternidade onde será feito o parto e a realização de atividades de educação em saúde e dos exames para propiciar o pré-natal adequado no município estudado. Além disso, os fatores associados à adequação devem ser levados em conta por parte dos gestores e profissionais de saúde.

## INTRODUCTION

Health evaluation has become an important and indispensable tool for the planning and management of services. Specifically in prenatal care, it should be emphasized that the results obtained by the evaluation may support both the maintenance of the strategies and their modification, with a view to improving the quality of care^1–3^.

In the specialized literature, we find the use of some procedures to evaluate prenatal care. Among these, the Kessner index^4^ and the Adequacy of Prenatal Care Utilization (APNCU), proposed by Kotelchuck^5^, stand out, which use as evaluative criteria the onset gestational age and the number of prenatal consultations^4–7^.

However, it is already known that these methods are insufficient to evaluate prenatal care, since they analyze only two aspects, preventing the visualization of relevant impacts on the quality of care. Therefore, it is necessary to insert new components that measure it integrally^8–10^.

In Brazil, the Ministry of Health, through the institution of the Prenatal and Birth Humanization Program (PHPN) and the *Rede Cegonha* Initiative, establishes guidelines for prenatal follow-up, guaranteeing the quality of care provided to pregnant women served in the public care network. In addition to the onset of prenatal care in the first trimester and the minimum number of seven appointments, laboratory tests and clinical-obstetric procedures are recommended, in addition to educational activities, immunization, multiprofessional care and guidelines on breastfeeding and childbirth^3,11–16^.

Despite the increase in prenatal coverage in the country, regional inequalities still persist. Specifically in the Northeast region, in recent national studies, there were lower coverage percentages, late prenatal onset, more difficulties in access and less examinations, as well as higher rates of maternal and neonatal deaths, which are related to low quality prenatal care^17^ ^,^ ^18^.

In the execution of prenatal care, Brazilian municipalities are responsible for coordinating the primary health care network. It presents itself as a gateway to the attention system for pregnant women and plays a fundamental role in the integral care of the mother-child binomial, providing better birth outcomes^13^.

In this sense, considering the importance of evaluation as starting point for possible interventions in health practices and the municipality as manager of primary health care, this study aimed to evaluate prenatal care in a northeastern capital from elements the structure, work process and results of the assistance. In addition, it aimed to verify demographic, socioeconomic, reproductive and maternal morbidity variables as possible factors associated with prenatal adequacy.

## METHODS

This is a transversal epidemiological study, developed in the city of João Pessoa, capital of the state of Paraíba, located in the Northeast region of Brazil. This municipality has 192 primary health care units, distributed in five health districts (HD): HD-I (49 units), HD-II (40), HD-III (50), HD-IV (29) and HD-V (24). HD concentrates neighborhoods by location proximity.

Participants in the study were professionals from the primary care services and puerperal who had prenatal care at these units. Specific forms with structure, work process, socioeconomic, demographic data and prenatal care questions were used. The information was obtained from November 2015 to August 2016.

The data related to the structure and work process were collected by interview with the nursing professionals of the units, chosen for having the greatest technical-administrative knowledge about how the services work. In each unit, the nurse responsible for prenatal care was selected, totaling 130 professionals. There was no refusal of interview by the nurses.

The sample calculation for the inclusion of primary health services and users was based on the formula^19^ ^,^ ^20^:

$$n=\frac{z^{2}.p.q.N}{e^{2}(N–1)+z^{2}.p.q}$$

For services, *z* is the standard normal distribution score (1.96) for a significance of 5% (or a 95% confidence); *p* refers to the proportion of health units with adequate care (as there is no reference parameter to estimate the comparability of representation of the target population, 50% was considered); *q* is the complement of the probability of occurrence of p (q = 1 - *p*)*, and* it is the margin of error (0.05); and *N,* the universe of units (192). With this calculation, the sample resulted in 130 units, distributed according to the proportion of the number of services of each health district in relation to the total units of the municipality: HD-I (32), HD-II (27), HD-III (34), HD-IV (19) and HD-V (18). The choice of units was performed randomly, using the statistical software R (version 2.10.1).

For the sample of users, the total population of live births of the municipality in the previous year in each health district was considered as *N* (HD-I: 2,557; HD-II: 2,011; HD-III: 3,319; HD-IV: 1,654; and HD-V: 2,330). This calculation resulted in the following sample: HD-I (340), HD-II (328), HD-III (352), HD-IV (317) and HD-V (336) with a total of 1,673 women. It should be noted that a safety margin of 20% was added to the calculation.

Patient data were collected from the municipal maternity hospital, Cândida Vargas, which accounts for the highest number of live births in the municipality, representing a percentage of 60.3% of all births in the capital in the year prior to the survey. The following were excluded from the study: women who did not had prenatal visits in João Pessoa, PB, or did not perform care in primary health care.

The collections occurred within 24 to 48 hours after birth. The women, from the sample units visited, were randomly selected to complete the sample from each health district. At that moment, to minimize the recall bias, the data were collected from the pregnant woman’s card (official document considered a valid and safe source of information for scientific research^21^).

The 48 users (2.9% of the sample) who did not present the pregnant woman’s card at the time of admission were considered as losses. Information from these women was collected through interviews; however, to avoid bias in the survey, they were not counted in the analyzes, resulting in a final sample of 1,625 users, which did not affect the representativeness of the municipality.

The research team was attended by nutrition students from the Federal University of Paraíba pre-selected by interviews and curricular analysis. All of them the were trained by the technical coordination of the research, through a previous training addressing the topics related to the study and the instruments used.

A pilot study was carried out, aiming to know the routine of the service, to test the instruments of collection and to experience the flow of the practice. After the collections, the questionnaires were reviewed and coded, with data entered into the Excel^®^ computer program with double entry for concordance evaluation and error checking. The errors, when detected, were solved by returning to the questionnaire or to the interviewer for correction of the database.

Prenatal care was classified by the IPR/Prenatal instrument regarding the aspects of infrastructure, process and results, as shown in the Box. This index is based on the Donabedian theoretical reference^7^ ^,^ ^22^ and has as evaluation criteria the recommendations of the national health authorities^11–13^. For it, for each of the questions of infrastructure, process and result analysis is assigned the value 1, when in accordance with the established recommendations, and 2 when not. Prenatal care is classified based on the percentage of the number of appropriate items in all components in relation to the total number of questions. Thus, prenatal care is classified according to the adequacy percentage obtained: adequate superior, when 100% of the items were adequate; adequate, when 75% or more were adequate; intermediary, when 51% to 74% of the answers were adequate; and inadequate, when it presented 50% or less of the criteria evaluated in accordance with the proposed recommendations.

**Box:** Classification of prenatal care by IPR/Prenatal. João Pessoa, state of Paraíba, Brasil, 2016.

Aspect	Evaluation criteria	Description of the criteria	Grade	Collection form
Infrastructure (services)	Construction in own building	Unit built with physical plant to be health service	1 (Yes) = adequate; 2 (No) = inadequate	Interview
	Exposure of health unit operation to users	Present in a visible place the days, shifts and prenatal professionals	1 (Yes) = adequate; 2 (No) = inadequate	Direct observation
	Equipment for prenatal use in operation^a^	To have at least six: clinical and Pinard stethoscope, sphygmomanometer, inelastic tape measure, glucometer, clinic table and chairs, stretcher, two-step iron ladder, scale, doppler fetal monitor/sonar, obstetric disk and exclusive cabinet for storage of medicines	1 (Yes) = adequate; 2 (No) = inadequate	Interview and direct observation
	Prenatal routine procedure materials	To have at least seven: exam request card, pregnant woman’s card, daily record map, referral form, medication prescription, follow-up form/handbook, disposable gloves, clean sheets for gynecological tables, materials for cytological collection, garbage basket, soap for personal hygiene, paper towel, gel alcohol, sink and toilet inside the office	1 (Yes) = adequate; 2 (No) = inadequate	Interview and direct observation
	Therapeutic supplies (medicines, quick tests, supplements, vaccines for prenatal use) ^b^	To have at least eight: ferrous sulfate, folic acid, hepatitis B vaccine, diphtheria and tetanus (dT), diphtheria, tetanus and pertussis (dTpa) vaccine, influenza vaccine, antacids, antibiotics, antipyretics, anthelmintics, complex B, vitamin C, hypotensives, antiemetics, anti-inflammatories and quick tests (HIV, syphilis and pregnancy)	1 (Yes) = adequate; 2 (No) = inadequate	Interview and direct observation
	Laboratory Support	Guarantee of laboratory support to perform the recommended examinations^c^	1 (Yes) = adequate; 2 (No) = inadequate	Interview
	Human Resources	Have the minimum team of primary health care^d^	1 (Yes) = adequate; 2 (No) = inadequate	Interview
Process (services)	Coverage of pregnant women followed up	Provide coverage of 100% of the pregnant women monitored in relation to the total area covered in the month prior to the visit	1 (Yes) = adequate; 2 (No) = inadequate	Note on documents in the units
	Coverage of pregnant women who met the goals of the Ministry of Health	To present coverage of 100% of the pregnant women who performed at least seven visits beginning in the first trimester and all the exams in the month prior to the visit in relation to the total number of pregnant women followed up (including immunization)	1 (Yes) = adequate; 2 (No) = inadequate	Note on documents in the units
	Multiprofessional service	Performing prenatal care with the presence of more than one senior professional	1 (Yes) = adequate; 2 (No) = inadequate	Interview
	Clinical-obstetric procedures	Team performs all the recommended procedures^e^	1 (Yes) = adequate; 2 (No) = inadequate	Interview
	Presence of clinical information on the follow-up of pregnant women	Team presents the same records on the pregnant woman’s card for the individual monitoring of users	1 (Yes) = adequate; 2 (No) = inadequate	Note on documents in the units
	Prescription of clinical exams	Prescription of recommended exams by professionals^c^	1 (Yes) = adequate; 2 (No) = inadequate	Interview
Continue				
**Box.** Classification of prenatal care by IPR/Prenatal. João Pessoa, state of Paraíba, Brasil, 2016. Continuation				
Results (users)	Orientação sobre aleitamento materno e sinais e sintomas do parto	To have received guidance on management, duration, possible breastfeeding problems, complementary feeding and birth	1 (Yes) = adequate; 2 (No) = inadequate	Interview
	Health Education	To have participated in prenatal health education activities	1 (Yes) = adequate; 2 (No) = inadequate	Interview
	Supplementation	Use of iron and folic acid during gestation	1 (Yes) = adequate; 2 (No) = inadequate	Interview
	Onset of prenatal care	To start prenatal care until the 12th gestational week	1 (Yes) = adequate; 2 (No) = inadequate	Pregnant woman’s card
	Number of medical consultations	To have had at least seven prenatal visits	1 (Yes) = adequate; 2 (No) = inadequate	Pregnant woman’s card
	Perform laboratory exams	To have performed the exams according to the Ministry of Health^c^	1 (Yes) = adequate; 2 (No) = inadequate	Pregnant woman’s card
	Immunization^f^	To have taken hepatitis B, tetanus, and influenza vaccines	1 (Yes) = adequate; 2 (No) = inadequate	Pregnant woman’s card
	Referral to maternity	To have received the referral to maternity hospital from primary care	1 (Yes) = adequate; 2 (No) = inadequate	Interview

Source: Brasil, 2000^11^; Silva et al.^7^, 2013.

^a^ Units where there was not doppler fetal monitor or sonar, or it was not functioning, were considered inadequate in the equipment aspect.

^b^ The isolated absence of ferrous sulfate, folic acid or vaccines recommended for gestation was considered inadequate.

^c^ Recommended exams: blood count (at least 2, fasting blood glucose: 2, blood typing: 1, Venereal Disease Research Laboratory (VDRL/Syphilis): 2, anti-HIV test: 2, toxoplasmosis: 1, hepatitis B and C: 2, urine summary: 2, ultrasonography: 1, electrophoresis: 1, Oral Glucose Tolerance Test (OGTT) and preventive cervical cancer if necessary.

^d^ Physician, nurse, nursing technicians, community health agents, dentist and auxiliary or oral health technician.

^e^ Registry of fetal movements, calculation of the probable date of birth, obstetric palpation, preventive cervical cancer test if necessary, assessment of nutritional status, measurement of uterine height, auscultation of the fetal heart rate, verification of blood pressure and edema, request of the exams and analysis of the breasts.

^f^ Specifically in relation to tetanus and hepatitis B, it was considered appropriate when the woman had the complete vaccination schedule, even if immunization occurred prior to gestation.

For this study, two evaluative criteria of the original instrument were not included, the gestational weight gain and puerperal consultation, since the data were obtained in a single moment in the postpartum period, without accompanying the woman during the prenatal and puerperium period.

The characteristics of the study population and prenatal care were presented in absolute and relative frequency distribution. The independent variables of the analysis included socio-demographic and economic characteristics: health district where the woman was assisted, age (≤ 18, 19–29 and ≥ 30 years old), living with the partner, *per capita* family income (considered as continuous variable), schooling (0–9 and ≥ 10 years), to be a beneficiary of the *Bolsa Família* program, and not be working during pregnancy.

The categorization of the age followed the parameters of the Child and Adolescent Statute, which considers the adult age group above 18 years old^23^. Schooling, in turn, followed the criteria adopted by the Basic Guidelines Law, which defines Brazilian education at levels: fundamental education, lasting nine years, and secondary and higher education, with 10 years or more of study. The categorization follows authors specialized in the theme, nationally and internationally^14^. For the categorical variables (health district, age and schooling), HD-I, the largest age range (≥ 30 years) and the lowest level of schooling (primary or primary education) were respectively considered as reference for the analyzes^15^ ^,^ ^23^ ^,^ ^24^. Reproductive (being primiparous and no abortions and premature births) and morbidities (diabetes, arterial hypertension, non-use of cigarettes and non-use of alcohol) characteristics were still considered.

Then, to verify the association of the independent variables with the prenatal adequacy, the logistic regression method was used based on the *odds ratio*, considering their respective confidence intervals (95%CI). For the analysis of the logistic regression, the prenatal classification was coded as “0” for “inadequate prenatal” (when classified as intermediate or inadequate), or “1” for “adequate prenatal” (adequate superior and adequate). The dependent variable considered for the study analyzes was “adequate prenatal.” Logistic regression was performed considering only the adequacy of the item “Results” due to the association with the independent variables used.

For the general model, all independent variables were analyzed with the dependent variable. From the stepwise technique, the inclusion and elimination of the independent variables were tested according to the significance power of each one in the analyzed outcome. The variables with the highest level of significance (p < 0.20) were inserted in the final model. To better explain the studied relationship, the quality-of-fit tests of the Nagelkerke R2 and Hosmer-Lemeshow final models^25^ ^,^ ^26^ were analyzed. In this model, the results were considered statistically significant at p < 0.05. Data were exported and analyzed in the SPSS application, version 20.0 (SPSS Inc., Chicago, IL, 2011)

Regarding the ethical aspects, the health and maternity units participated in the study by signing the letter of agreement of the Municipal Health Department. The professionals and users participated after signing the free and informed consent form and the free and informed consent term. The research was approved by the Ethics and Research Committee of the Lauro Wanderley University Hospital of the Federal University of Paraíba under the number 381335414.7.0000.5183.

## RESULTS

Regarding the characteristics of the health services (Table 1), it can be observed that most of the units were located in places specifically built for this purpose and had visible days, shifts and professionals who carried out the prenatal care. The equipment was in operation and there were vaccines, medicines and important supplements for prenatal care in about 70% of the services. The presence of reference laboratory support was reported by 100% of the professionals, while the minimum primary care team was observed in almost 90% of the units.

**Table 1:** Characterization of prenatal services and users of primary care in João Pessoa, state of Paraíba, Brazil, 2016.

Characterization of health services		n	%
Construction in own building		105	80.8
Knowledge of users about the health unit’s functioning		125	96.2
Equipment in operation		92	70.8
Materials of prenatal routine procedures		126	96.9
Vaccines, medications and supplements		97	66.9
Reference laboratory support		130	100.0
Human Resources		115	88.5
Coverage of pregnant women followed up		120	92.5
Coverage of pregnant women who met the goals of the Ministry of Health		38	29.1
Multiprofessional service		110	84.7
Presence of clinical information on the follow-up of pregnant women		130	100.0
Performing clinical-obstetric procedures		130	100.0
Prescription of clinical exams		130	100.0

Characterization of users	n	%	Mean (SD)

Guidance on the type of birth	773	47.6	
Guidance on childbirth symptoms	765	47.1	
Guidance on breastfeeding	751	46.2	
Participation in health education activities	406	25.0	
Use of supplementation during pregnancy	1,279	78.7	
Start on the first trimester	858	52.8	14.30 (4.55) gestational weeks
Number of medical consultations			
< 7	648	39.9	5.86 (1.35) medical consultations
≥ 7	977	60.1	
Performing the exams recommended in prenatal care	214	13.2	
Immunization	1,154	71.0	
Referral to maternity	444	27.3	

Regarding the characterization of the work process, there was a high coverage of pregnant women followed up by the units. However, when the number of women who started prenatal care in the first trimester, with more than seven visits and who performed the recommended exams, was analyzed, a small part of the prenatal services were able to meet these parameters.

In most services, more than one professional of higher level (at least one doctor and one nurse) was present in prenatal care. The total number of professionals reported the presence of the clinical records of pregnant women, referred to perform all the recommended clinical-obstetric procedures and prescribe the clinical exams.

Regarding the characteristics of the users, in relation to prenatal care (Table 1), less than half of the women were guided on the type and symptoms of childbirth and on breastfeeding. Only 25% participated in prenatal educational activities.

The number of women who used iron and folic acid supplements during pregnancy, who had seven or more visits, and had prenatal care in the first trimester increased. Regarding immunization, there was a coverage of 71% for the complete vaccination scheme for gestation. It stands out the low percentage of women who underwent the recommended examinations (13.4%) and women who received the referral to the maternity hospital (27.3%). When classifying prenatal care using the IPR/Prenatal care criteria, the municipality of João Pessoa showed adequacy in only 22.6% of cases (Table 2).

**Table 2:** Qualification of prenatal care according to aspects of structure, process and results in primary care.

Categorization	Classification		95%CI

	n	%	
Adequate	367	22.6	19.2–26.0
Intermediary	278	17.1	14.0–20.3
Inadequate	980	60.3	50.0–70.6

Table 3 shows the characteristics of the users regarding the adequacy of prenatal care, which was higher in women between 19 and 29 years old, with 10 years of schooling or more, with a family income greater than a minimum wage, who did not work during pregnancy and living with a partner. It was also observed that beneficiaries of *Bolsa Família* program, primiparous women, who did not have previous abortions and preterm infants, did not drink alcohol, did not smoke and did not have diabetes, hypertension and edema during pregnancy presented a higher percentage of adequate prenatal care.

**Table 3:** Characterization of the users according to the prenatal classification by IPR/Prenatal. João Pessoa, state of Paraíba, Brasil, 2016.

Independent variables	Total women interviewed		Adequate prenatal care		Inadequate prenatal care		p*

	(n = 1,625)		n = 367 (22.6%)		n = 1,258 (77.4%)		

	n	%	n	%	n	%	
Health district							0.723
I	330	20.3	66	18.0	264	21.0	
II	318	19.6	66	18.0	252	20.0	
III	345	21.2	68	18.5	277	22.0	
IV	307	18.9	54	14.7	253	20.1	
V	325	20.0	113	30.8	212	16.9	
Age (years old)							< 0.001
≤ 18	73	4.6	8	2.2	65	5.2	
19–29	1,311	80.5	192	52.3	1,119	88.9	
≥ 30	241	14.9	167	45.5	74	5.9	
Level of education							< 0.001
0–9 years	1,067	65.6	38	10.3	1,029	81.8	
≥ 10 years	558	34.4	329	89.7	229	18.2	
Monthly average income							< 0.001
≤ 1 MW	372	22.8	127	34.6	245	19.5	
> 1 MW	1,253	77.2	240	65.4	1,013	80.5	
Work in Pregnancy							< 0.001
Yes	1,001	61.6	100	27.2	901	71.6	
No	624	38.4	267	72.3	357	28.4	
Lives with a partner							< 0.001
Yes	1,093	67.3	324	88.3	769	61.1	
No	532	32.7	43	11.7	489	38.9	
Enrollment on the *Bolsa Família* Program							< 0.001
Yes	517	31.8	241	65.7	276	22.0	
No	1,108	67.8	126	34.3	982	78.0	
Parity							< 0.001
Primiparous	465	28.6	307	83.7	158	12.5	
Multiparous	1,160	71.4	60	16.3	1,100	87.5	
Previous abortions							< 0.001
Yes	691	42.5	24	6.5	667	53.0	
No	934	54.5	343	93.5	591	47.0	
Previous premature infants							< 0.001
Yes	520	32.0	67	18.3	453	36.0	
No	1,105	68.0	300	81.7	805	64.0	
Intake of alcoholic beverage							< 0.001
Yes	565	34.8	32	8.7	533	42.4	
No	1,060	65.2	335	91.3	725	57.6	
Smoker							< 0.001
Yes	221	13.6	14	3.8	207	16.4	
No	1,404	86.4	353	96.2	1,051	83.6	
Diabetes							0.003
Yes	164	10.1	22	6.0	142	11.3	
No	1,461	89.9	345	94.0	1,116	88.7	
Arterial hypertension							< 0.001
Yes	161	9.9	19	5.2	142	11.3	
No	1,464	90.1	348	94.8	1,116	88.7	
Presence of edema							< 0.001
Yes	869	53,5	68	18.5	801	63.4	
No	756	46.5	299	81.5	457	36.4	

MW: minimum wage (at the time of the study, it was equivalent to R$880.00 or US$281.12).

* Chi-square test.

Table 4 shows the analyzes of the independent variables with prenatal adequacy. After the adjusted model, it was seen that women between 19 and 29 years old, with more years of study, higher family income and primiparous had more chance of adequate prenatal care. Not having previous abortions did not show statistical significance in the adjusted model. It stands out the increase of the R^2^ measure of the models from 0.425 to 0.708, indicating that the adjusted model is approximately 71% sure that these factors are related to the prenatal adequacy, guaranteeing the confidence of the analyzes.

**Table 4:** Adjusted logistic regression of the variables with the adequacy of prenatal care in primary care.

Variable	Gross OR	95%CI	Adjusted OR	95%CI
Health district				
District I	1			
District II	0.750	0.157–1.646		
District III	0.820	0.490–1.741		
District IV	0.720	0.230–1.950		
District V	1.400	0.960–1.620		
Age (years old)				
≤ 18	1.260	0.870–1.719	0.820	0.320–1.720
19–29	2.720	1.090–3.140	1.390	1.120–2.220
≥ 30	1		1	
Level of education				
0–9 years	1			
≥ 10 years	2.520	1.480–3.160	1.750	1.156–2.233
Monthly average income			1	
≤ 1 MW	1			
> 1 MW	2.150	1.390–3.260	1.870	1.114–2.420
Work in Pregnancy				
Did not work in pregnancy	0.860	0.665–1.054		
Worked in pregnancy	1			
Lives with a partner				
Yes	0.779	0.674–1.459		
No	1			
Enrollment on the *Bolsa Família* Program				
Yes	0.793	0.623–1.079		
No	1			
Parity				
Primiparous	1.335	1.140–2.020	1.230	1.100–1.879
Multiparous	1		1	
Previous abortions				
No	1.175	0.865–1.596	0.980	0.806–1.020
Yes	1		1	
Intake of alcoholic beverage				
No	1.062	0.758–1.206		
Yes	1			
Smoker				
No	1.042	0.914–1.264		
Yes	1			
Diabetes				
No	2.260	0.896–8.059		
Yes	1			
Arterial hypertension				
No	1.036	0.548–1.753		
Yes	1			
Presence of edema				
No	0.870	0.610–1.298		
Yes	1			
Previous premature infants				
No	1.430	0.936–2.325		
Yes	1			

MW: minimum wage (at the time of the study, it was equivalent to R$880.00 or US$281.12).

R^2^ not adjusted: 0.425; R^2^ adjusted: 0.708.

Unadjusted Hosmer and Lemeshow test: 0.475; adjusted Hosmer and Lemeshow test: 0.778.

## DISCUSSION

The evaluation of prenatal care from the triad structure, work process and result allows to identify more accurately factors that contribute to the improvement of health practices, seeking the qualification of care^5^ ^,^ ^7^ ^,^ ^9^ ^,^ ^22^. In this context, regarding the aspects related to the structure and the work process, it was observed a frequency below what was considered adequate (75%) for the presence of equipment, therapeutic supplies and coverage according to the goals proposed by the Ministry of Health. The presence of equipment, therapeutic supplies and laboratory support enoough to meet the demand favors the performance of prenatal care, since it guarantees the necessary procedures and interventions with resolutive actions^15^.

Regarding the prenatal care of the studied municipality, low prevalences for educational strategies and guidance throughout care were observed. When developed in a continuous and participative way throughout the prenatal period, from the team’s dialogue with the user, they contribute to better obstetric outcomes. The sensitivity of breastfeeding practice to educational actions stands out: mothers who participated in health education strategies had a longer breastfeeding period^27^.

Regarding the onset of prenatal care and number of consultations, although the studied municipality showed a greater number of women with onset in the first trimester and with seven or more visits, the prevalence was below the adequate. Prenatal care in early pregnancy has as a great advantage the early detection of possible complications during pregnancy and the guarantee of timely interventions^13^. On the other hand, the lower number of consultations is associated with less adequacy of exams, vaccination and guidance on breastfeeding and childbirth^15^.

One finding that deserves attention is the small number of women who underwent laboratory tests, as observed in other studies^3^ ^,^ ^7^ ^,^ ^15^. Performing the exams during the gestational period is important to prevent possible problems that can be previously solved in prenatal care. Polgliane et al.^3^ point to several potential difficulties that justify this low prevalence, mainly due to the organization of the health services, including difficulties in scheduling exams, lack of inputs for its performance and malfunctioning equipment. These complications hamper adequate time to return the results to necessary interventions.

Only a small percentage of women were referred to maternity care by primary care services. In Brazil, pregnant women attending the Unified Health System have the right to be linked to the maternity hospital where they will receive childbirth care, which must be guaranteed from the beginning of prenatal care. The omission may lead to a pilgrimage by the health facilities at the time of birth, which may favor the occurrence of maternal and child morbidity and mortality in the country^2^.

Regarding the evaluation of care, as well as in other studies that used the same instrument as this research, a low prevalence of adequacy was observed^7^ ^,^ ^28^. The adequacy is the result of the satisfactory result of several components that need to be considered in the evaluation process, such as: structure and access to services, presence of equipment and supplies in the units, available human resources, assurance of exams and education activities in health, among other aspects^7^ ^,^ ^15^.

Women with favorable socioeconomic, reproductive and morbidity conditions had a higher percentage of prenatal adequacy. These findings are corroborated by other researchers who identified a lower percentage of adequate prenatal care in more vulnerable populations^15^ ^,^ ^24^. The analyzes confirmed the presence of these social inequities during prenatal care, specifically regarding socioeconomic conditions. It was observed that being an adult, with more years of study and higher *per capita* income were factors associated with prenatal adequacy. The studies warn that managers and prenatal care teams need to be prepared to work to alleviate this difference, putting into practice the principle of social equity^15^ ^,^ ^16^ ^,^ ^23^ ^,^ ^24^ ^,^ ^29^.

The adequacy of prenatal care was also determined by reproductive variables such as parity. In this sense, researchers affirm that primiparity may favor the qualification of prenatal care, once multiparous women tend not to perform prenatal care in a regular way, since, from previous experience, they believe they already know about the course of gestation, its intercurrences and the breastfeeding practice^30^.

Based on the diagnosis made, the prenatal evaluation by an instrument that incorporates broader criteria in its analysis – with aspects of infrastructure, work process and result – allowed to verify more appropriately the actual situation of assistance. With the application of this index, the municipality studied presented low percentage of adequacy, determined by factors that should be discussed by the family health teams during the development of care.

From this perspective, regarding socioeconomic conditions, the construction of public policies aimed at reducing the inequities that also guide prenatal care should be strengthened. As for the reproductive aspects, the health team should increase the intake of pregnant women, making consultation schedules more flexible so that mothers with more children also have adequate prenatal care.

It is higlighted that the small number of women who were referred to the maternity hospital participated in health education activities and performed the exams recommended for prenatal care. It is worth noting that the number of women who started prenatal care in the first trimester and the highest number of consultations, although corresponding to more than half of the women, also remained below expectations. Therefore, strategies that ensure and stimulate these procedures should be considered by managers and health teams during prenatal care, to ensure adequate and resolute care.

As a possible limitation of the study, there is the non inclusion of women attended in other hospitals. However, it is worth mentioning that the analyzed maternity hospital is the highest reference for childbirth in the municipality, with the highest number of visits.

For the next studies, it is recommended to include the users in the observations of the work process and the reproduction of the instrument in other places, contributing to the knowledge of the prenatal care reality and to the elaboration of possible interventions in case of non-compliance with the recommended guidelines.
